# A passive mutualistic interaction promotes the evolution of spatial structure within microbial populations

**DOI:** 10.1186/s12862-017-0950-y

**Published:** 2017-04-24

**Authors:** Marie Marchal, Felix Goldschmidt, Selina N. Derksen-Müller, Sven Panke, Martin Ackermann, David R. Johnson

**Affiliations:** 1 Department of Environmental Microbiology, Eawag, Überlandstrasse 133, 8600 Dübendorf, Switzerland; 2 Department of Environmental Systems Science, ETH Zürich, 8092 Zürich, Switzerland; 3 Department of Biosystems Science and Engineering, ETH Zürich, 4058 Basel, Switzerland

**Keywords:** Mutualism, Cross-feeding, Cell aggregation, Experimental evolution, Microbial populations, Spatial structure

## Abstract

**Background:**

While mutualistic interactions between different genotypes are pervasive in nature, their evolutionary origin is not clear. The dilemma is that, for mutualistic interactions to emerge and persist, an investment into the partner genotype must pay off: individuals of a first genotype that invest resources to promote the growth of a second genotype must receive a benefit that is not equally accessible to individuals that do not invest. One way for exclusive benefits to emerge is through spatial structure (i.e., physical barriers to the movement of individuals and resources).

**Results:**

Here we propose that organisms can evolve their own spatial structure based on physical attachment between individuals, and we hypothesize that attachment evolves when spatial proximity to members of another species is advantageous. We tested this hypothesis using experimental evolution with combinations of *E. coli* strains that depend on each other to grow. We found that attachment between cells repeatedly evolved within 8 weeks of evolution and observed that many different types of mutations potentially contributed to increased attachment.

**Conclusions:**

We postulate a general principle by which passive beneficial interactions between organisms select for attachment, and attachment then provides spatial structure that could be conducive for the evolution of active mutualistic interactions.

**Electronic supplementary material:**

The online version of this article (doi:10.1186/s12862-017-0950-y) contains supplementary material, which is available to authorized users.

## Background

**Fig. 1:**
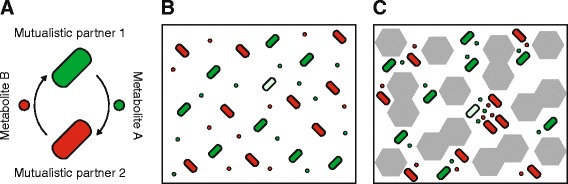
Spatial structure promotes the evolution of active metabolite excretion. **a** A mutualistic interaction between two mutualistic partners. Partner 1 (*green cell*) excretes metabolite A (*green circle*) that promotes the growth of partner 2. Partner 2 (*red cell*) excretes metabolite B (*red circle*) that promotes growth of partner 1. **b** In a well-mixed environment, a mutant green cell that actively excretes more metabolites (*light green cell*) should decrease in frequency. This is because the mutant cell would pay all of the cost for increased metabolite excretion but would receive the same benefit as all the other green cells. **c** In a spatially structured environment, a mutant green cell that actively excretes more metabolites (*light green cell*) could potentially increase in frequency. This is because spatial structure might cause the mutant cell to receive a disproportionate amount of the benefits from increased metabolite excretion. These benefits originate from the positive effects of increased excretion of metabolite A on partner 2, thus resulting in increased excretion of metabolite B

Mutualistic interactions are pervasive in the natural environment and shape the assembly and functioning of nearly every ecological community [1, 2]. A mutualistic interaction occurs when two (or more) different organisms – referred to here as mutualistic partners - have reciprocal positive effects on each other’s growth. Examples of mutualistic interactions include the associations between legumes and nitrogen-fixing bacteria, between plants and pollinating insects, and between humans and members of their gut microbiota [1, 2]. Mutualistic interactions are also common between members of microbial communities and are important determinants of their ecological dynamics and processes [3]. A typical feature of mutualistic interactions within microbial communities is that they often require one or both mutualistic partners to excrete metabolites that positively affect the growth of others [4–8] (Fig. 1a). While we mainly focus on this type of mutualistic interaction in this manuscript, the main idea that we develop is potentially relevant for other types of mutualistic interactions and organisms.

How mutualistic interactions emerge and persist is not clear [9–11]. A first question pertains to the origin of mutualistic interactions. Starting from a situation where two microbial genotypes exist in the same environment but do not affect each other’s growth, how could a mutant of one genotype emerge that excretes a metabolite that positively affects the growth of another genotype and increases in frequency, thus laying the foundation for a mutualistic interaction to evolve? In this scenario, the mutant might not receive an immediate benefit but would potentially carry a metabolic cost associated with excreting the metabolite. The mutant might therefore not be able to increase in frequency relative to its ancestor and a mutualistic interaction could not establish.

One possible solution to this problem is that mutually beneficial interactions could originally be *passive* in nature [12, 13]. We use the term ‘passive’ to refer to a behavior or property of an organism that, while potentially being beneficial to another organism, did not evolve because of its positive effects on that other organism. This is particularly evident for metabolic interactions between microorganisms, which often passively excrete metabolites that positively affect the growth of other microorganisms [12, 14–16]. For example, microorganisms might excrete metabolites via cell leakage or as side-products or end-products of their own metabolism [16–19], which could then be taken up by other microorganisms. If such metabolic interactions are reciprocal between two partners (Fig. 1a), they constitute as a mutually beneficial but passive mutualistic interaction.

While the passive scenario described above could be important for the origin of a mutualistic interaction, many mutualistic interactions in nature are not passive but are rather based on the *active* excretion of metabolites that positively affect the growth of a mutualistic partner (e.g. [7]). The active excretion of metabolites must, at least to some extent, divert cellular resources away from the growth and reproduction of the excreting microorganism [20–22]. These metabolic costs then lead to a second fundamental question: starting from a situation where two microorganisms are coupled by an initially passive mutualistic interaction, how could investment of metabolic resources into each mutualistic partner evolve? Such an evolutionary transition requires a mutant that actively excretes metabolites to increase in frequency. Again, this is not trivial to explain. A mutant that actively excretes metabolites would increase the growth of its mutualistic partner, which in turn would lead to a benefit that is accessible to both the mutant and its ancestor (Fig. 1b). While the benefits of an increased investment are thus distributed uniformly, the costs are borne alone by the actively excreting mutant. The mutant might therefore have a growth disadvantage relative to its ancestor and decrease in frequency.

Ultimately, the evolutionary transition of a passive into an active mutualistic interaction requires that the mutant that actively invests metabolic resources into the growth of its mutualistic partner receive an exclusive benefit [9, 23]. Such coupling between investment and return arises if individuals of different mutualistic partners are spatially associated with each other for long periods of time [24, 25]. In this case, a mutant that invests metabolic resources into the growth of its mutualistic partner would receive an increased return if the partner’s growth leads to an increased production of the benefit. This situation has been referred to as the “partner-fidelity model” [24]. A general scenario under which such a long-term association between individuals could arise is in the presence of spatial structure [11, 20, 21, 23, 25–29] (Fig. 1c); that is, in a situation where physical barriers constrain the movement of individuals and metabolites. In spatially structured environments, the benefits arising from an investment into another organism are not distributed evenly but are instead disproportionally directed back towards the investor, thus allowing those individuals to potentially increase in frequency and spread [20, 21, 23, 26–29] (Fig. 1c).

While there is accumulating theoretical and experimental evidence to support the importance of spatial structure on the evolution of mutualistic interactions, these studies have largely focused on imposing abiotic spatial structure on a mutualistic consortium and analyzing the evolutionary outcomes (e.g. [20, 21, 26, 30]). Mutualistic interactions, however, are also observed in habitats with relatively little abiotic spatial structure, for example between microorganisms that reside within the water columns of open oceans and lakes (e.g. [7, 31]). This then underscores an important gap in our knowledge: How can we explain the evolution of an active mutualistic interaction in the absence of extensive abiotic spatial structure?

The central idea that we are addressing in this manuscript is that microorganisms can readily evolve to create their own spatial structure based on physical attachment between individuals (i.e., cell aggregation) [23]. More specifically, we test the hypothesis that an initially passive mutualistic interaction selects for mutants that aggregate together with other individuals and thereby benefit from increased local concentrations of excreted metabolites. This scenario could have important consequences because cell aggregation leads to “partner fidelity” and could set the stage for the evolution of an active mutualistic interaction. Indeed, cell aggregates are prevalent within the mixed layers of oceans and non-stratified lakes and can harbor mutualistic interactions (e.g., [7, 31]). Whether a passive mutualistic interaction itself could promote the evolution of cell aggregation, however, is not clear.

To test this hypothesis, we experimentally created a passive mutualistic interaction between two auxotrophic strains of the bacterium *Escherichia coli* and tested for the evolution of cell aggregation. Each strain is defective in the biosynthesis of a different amino acid; they can only grow if the required amino acid is exogenously supplied from an abiotic source or if the strains are grown together in co-culture and passively excrete or release small amounts of the amino acid required by the other. This passive mutualistic interaction was based on a single genetic mutation in each mutualistic partner; it could therefore originate spontaneously via random mutation within large populations. We then propagated the mutualistic co-cultures in the absence of extensive abiotic spatial structure (i.e., in continuously-mixed batch reactors) and tested whether the passive mutualistic interaction itself promotes the evolution of cell aggregation. We are not investigating how spatial structure influences the evolutionary transition to an active mutualistic interaction in this manuscript. Instead we ask how spatial structure itself evolves, and thereby focus on a process that has potentially profound implications for interactions both within and between populations of organisms.

## Methods

### Bacterial strains

We obtained amino acid-auxotrophic strains of *E. coli* BW25113 from the KEIO single-gene knockout library [32] (Table 1). Strain BW25113 (∆*proC*) contains a complete deletion of the open-reading frame of the *proC* gene and is predicted to be defective in proline biosynthesis. Strain BW25113 (∆*trpC*) contains a complete deletion of the open-reading frame of the *trpC* gene and is predicted to be defective in tryptophan biosynthesis. We refer to these strains as ‘mutualistic partners’ because they can grow together in co-culture but cannot grow alone; the ability to grow together is based on the passive excretion – presumably via cell leakage - of the amino acid that the other strain cannot biosynthesize.

**Table 1:** Bacterial strains and plasmids used in this study

Strain or plasmid	Relevant characteristics	Reference or source
*E. coli* strain		
BW25113 (∆*proC*)	BW25113 with ∆*proC*:*Km* ^*R*^; Km^R^	[32]
BW25113 (∆*trpC*)	BW25113 with ∆*trpC*:*Km* ^*R*^; Km^R^	[32]
DH5α/λpir	Used for replication of pUC18T derivatives; λ*pir*80*dlacZ *Δ*M15* Δ(*lacZYA-argG*)*U169 recA1 hsdr17 deoR thi-1 supE44 gyrA96 relA*	[34]
Plasmid		
pUC18T-mini-Tn7T-LAC-Gm	pUC18-based conditionally replicative delivery plasmid for mini-Tn7-LAC-Gm; Ap^R^, Gm^R^, mob^+^	[33]
pUC18T-mini-Tn7T-LAC-Gm-e*gfp*	pUC18T-mini-Tn7T-LAC-Gm containing e*gfp* immediately downstream of P_*lac*_; Ap^R^, Gm^R^, mob^+,^ egfp^+^	this study
pUC18T-mini-Tn7T-LAC-Gm-e*cherry*	pUC18T-mini-Tn7T-LAC-Gm containing e*cherry* immediately downstream of P_*lac*_; Ap^R^, Gm^R^, mob^+,^ echerry^+^	this study

We verified that the mutualistic partners are auxotrophic for the predicted amino acid by growing them in isolation with liquid minimal medium that was or was not supplemented with the required amino acid. The liquid minimal medium consisted of 6.8 g L^−1^ Na_2_HPO_4_ × 7H_2_O, 3 g L^−1^ KH_2_PO_4_, 0.5 g L^−1^ NaCl, 1 g L^−1^ NH_4_Cl, 3.6 g L^−1^ glucose, 0.24 g L^−1^ MgSO_4_, and 10 mg L^−1^ gentamycin (referred to as MM hereafter). We streaked each mutualistic partner onto a different lysogeny broth (LB) agar plate, picked three colonies from each LB agar plate, inoculated each colony into a different test tube containing 3 ml of MM, and incubated the test tubes for 24 h at 37 °C with continuous shaking (220 r. p. m.). As expected, neither mutualistic partner could grow in isolation with MM. However, strain BW25113 (∆*proC*) could grow in isolation when we supplemented MM with 50 mg L^−1^ L-proline while strain BW25113 (∆*trpC*) could grow in isolation when we supplemented MM with 20 mg L^−1^ L-tryptophan, thus verifying that each mutualistic partner is indeed auxotrophic for the predicted amino acid.

### Genetic manipulations

We introduced a different plasmid into each mutualistic partner that carries a gene encoding for a different florescent protein, thus allowing us to distinguish and individually quantify each mutualistic partner when they are grown together in co-culture. To accomplish this, we constructed two derivatives of the pUC18T-mini-Tn7T-LAC-Gm conditionally replicative plasmid (Table 1) [33]. This plasmid contains an isopropyl-β-D-thiogalactopyranosid (IPTG)-inducible P_*lac*_ promoter located immediately upstream of a multiple cloning site (MCS). We first purified the pUC18T-mini-Tn7T-LAC-Gm plasmid from an overnight culture of *E. coli* DH5α/λpir (Table 1) [34]. We next used GoTaq DNA polymerase (Promega, Madison, WI, USA) to PCR amplify the *egfp* or *echerry* gene [35], which encode for green or red fluorescent protein respectively. The PCR amplification primers contain the *Bam*HI and *Kpn*I restriction sites that we used to clone the PCR products into the MCS of the pUC18T-mini-Tn7T-LAC-Gm plasmid (See Additional file 1). We then digested the PCR products and the pUC18T-mini-Tn7T-LAC-Gm plasmid with *Bam*HI and *Kpn*I (Thermo Fisher Scientific, Waltham, MA, USA) and ligated the PCR products into the pUC18T-mini-Tn7T-LAC-Gm plasmid. We designated the assembled derivative plasmids as pUC18T-mini-Tn7T-LAC-Gm-*egfp* and pUC18T-mini-Tn7T-LAC-Gm-*echerry* (Table 1). We finally replicated the assembled derivative plasmids in *E. coli* DH5α/λpir (Table 1) [34], introduced the derivative plasmids into the mutualistic partners via electroporation, and selected for transformants carrying the derivative plasmids by plating on LB agar plates supplemented with 10 μg ml^−1^ gentamycin and 1 mM IPTG. We introduced each derivative plasmid into each mutualistic partner, resulting in both *egfp*- and *echerry*-expressing variants of strains BW25113 (∆*proC*) and BW25113 (∆*trpC*).

### Evolution experiment

We performed an evolution experiment with replicated co-cultures of the two mutualistic partners. We streaked the *egfp*-expressing BW25113 (∆*proC*), *echerry*-expressing BW25113 (∆*trpC*), *echerry*-expressing BW25113 (∆*proC*), and *egfp*-expressing BW25113 (∆*trpC*) strains onto different LB agar plates that were supplemented with 10 μg ml^−1^ gentamycin and 1 mM IPTG. We then picked one colony from each LB agar plate, inoculated each colony into a different test tube containing 2.7 ml of MM supplemented with 300 μl of liquid LB medium, and incubated the test tubes for 24 h at 37 °C with continuous shaking (220 r. p. m.). After reaching stationary phase, we centrifuged the cultures, discarded the spent medium, washed the cells twice with H_2_O containing 9 g L^−1^ NaCl, and suspended the washed cells in H_2_O containing 9 g L^−1^ NaCl. We next prepared two binary mixes (i.e. co-cultures) of the mutualistic partners (50:50 ratio based on optical density measurements at 600 nm [OD_600_]); one set of co-cultures consisted of the *egfp*-expressing BW25113 (∆*proC*) and *echerry*-expressing BW25113 (∆*trpC*) mutualistic partners while the other set of co-cultures consisted of the *echerry*-expressing BW25113 (∆*proC*) and *egfp*-expressing BW25113 (∆*trpC*) mutualistic partners. We finally inoculated 300 μl of each co-culture into eight replicated test tubes containing 2.7 ml of MM that was supplemented with 1 mM IPTG but not with amino acids, resulting in a total of 16 replicated mutualistic co-cultures. We designated the co-cultures consisting of the *egfp*-expressing BW25113 (∆*proC*) and *echerry*-expressing BW25113 (∆*trpC*) mutualistic partners as mutualistic co-cultures A1 to A8 and the co-cultures consisting of the *echerry*-expressing BW25113 (∆*proC*) and *egfp*-expressing BW25113 (∆*trpC*) mutualistic partners as mutualistic co-cultures B1 to B8. The initial OD_600_ of each mutualistic co-culture was approximately 0.12. After incubating the mutualistic co-cultures for 3.5 days at 37 °C with continuous shaking (220 r. p. m.), we transferred 300 μl of each co-culture to a new test tube containing 2.7 ml of fresh MM that was supplemented with 1 mM IPTG but not with amino acids to achieve a 1:10 (volume: volume) dilution. We then repeated the incubation and transfer steps in the same MM supplemented with 1 mM IPTG for a total of 16 serial transfers. Immediately before each transfer, we measured the OD_600_ of each mutualistic co-culture and archived a portion of each mutualistic co-culture in 20% glycerol at −80 °C for further analyses. We quantified the magnitude of cell aggregation within each mutualistic co-culture immediately before the fourteenth transfer as described below. If all cells in these co-cultures would have grown at the same rate, then the fourteen transfers with ten-fold dilution would correspond to approximately 46 generations of growth (14 × log_2_10); however, if only a portion of the cells would have grown efficiently under our experimental conditions (for example mutants that attach to other cells), then the number of cell generations during the evolution experiment could have been potentially much larger. We performed control experiments with the ancestral mutualistic partners using growth conditions identical to those described above, except that the MM was supplemented with 50 mg L^−1^ L-proline and 20 mg L^−1^ L-tryptophan.

### Quantification of cell aggregation

We imaged each mutualistic co-culture using a Leica TCS SP5 confocal laser-scanning microscope (CLSM) with a 63 × (1.4 NA) oil-immersion lens (Leica Microsystems, Wetzlar, Germany). We removed 5-μl liquid aliquots from each mutualistic co-culture immediately after removal from the shaking incubator and deposited the liquid aliquots onto the surface of a glass slide. We imaged *egfp*-expressing cells using 488 nm excitation wavelength and 500–530 nm emission wavelengths. We imaged *echerry*-expressing cells using 633 nm excitation wavelength and 657–757 nm emission wavelengths. We collected images at a resolution of 1024 × 1024 using LAS AF v2.7 software (Leica Microsystems).

We quantified cell aggregation using the StatColoc plugin of the Icy software [36]. This algorithm computes the two-dimensional co-localization of different objects (in our case cells) using the Ripley’s K function [37]. The resulting K-value measures the degree to which a set of objects deviates from spatial homogeneity. In our experiments with completely mixed batch reactors, a deviation from spatial homogeneity is most likely caused by cell aggregation, which we indeed confirmed by microscopy. We first detected *e* *gfp*- and *echerry*-expressing cells using the Spot Detector plugin of Icy and translated the images into binary data using the NIH ImageJ analysis software (http://rsbweb.nih.gov/ij/). We then applied the StatColoc plugin using a radius of 0.48 μm to 4.8 μm. We analyzed between five and nine randomly selected microscope fields for each mutualistic co-culture and obtained ten K-values as a function of distance for each microscope field. We finally identified the maximum observed K-value for each microscope field and tested whether the maximum observed K-values are significantly different between test and reference mutualistic co-cultures using the non-parametric Mann-Whitney U test. We performed the same statistical tests with the sum-of-the-ten K-values rather than the maximum observed K-value and obtained qualitatively identical results. We chose here to report the maximum observed K-values because they had better statistical properties. Namely, many of the microscopy fields did not contain any cell aggregates, which would be expected when aggregation is extensive and consists of a few sparsely distributed objects. This increases the variance when using the sum-of-the-ten K-values. We implemented the Mann-Whitney U test with the StatColoc plugin and considered a two-sided *P* < 0.05 to be statistically significant.

### Isolation of evolved mutualistic partners

We isolated mutants emerging within each archived mutualistic co-culture that remained viable immediately before the fourteenth transfer of experimental evolution. We first streaked each archived mutualistic co-culture onto different LB agar plates that were supplemented with 10 μg ml^−1^ gentamycin and 1 mM IPTG. In many cases, the colonies expressed multiple fluorescent proteins, and we therefore had to perform a second streaking. After obtaining single colonies that only express one fluorescent protein, we qualitatively distinguished different evolved mutualistic partners within each co-culture based on colony morphology and the fluorescent gene that they expressed (*egfp* or *echerry*). For some co-cultures, we identified more than one colony morphology for each fluorescent gene. We finally picked one colony for each morphology, inoculated each colony into a different test tube containing 3 ml of liquid LB medium supplemented with 10 μg ml^−1^ gentamycin and 1 mM IPTG, incubated the test tubes for 24 h at 37 °C with continuous shaking (220 r. p. m.), and archived a portion of each culture in 20% glycerol at −80 °C for further analyses.

### Genome sequencing of evolved mutualistic partners

We sequenced the genomes of all the isolated evolved mutualistic partners (see Additional file 2). In parallel, we sequenced the genomes of all the ancestral mutualistic partners (i.e., the *egfp*-expressing BW25113 (∆*proC*), *echerry*-expressing BW25113 (∆*trpC*), *echerry*-expressing BW25113 (∆*proC*), and *egfp*-expressing BW25113 (∆*trpC*) strains). We first streaked each mutualistic partner from the glycerol-archived samples onto different LB agar plates supplemented with 10 μg ml^−1^ gentamycin and 1 mM IPTG. We then picked one colony from each LB agar plate, inoculated each colony into a test tube containing 3 ml of liquid LB medium, incubated the test tubes for 24 h at 37 °C with continuous shaking (220 r. p. m.), and extracted genomic DNA from each culture using the ArchivePure DNA Purification kit (5prime, Hilden, Germany). We then prepared one sequence library for each mutualistic partner using 1 ng of genomic DNA, the Nextera XT DNA Sample Preparation kit (Illumina Inc., San Diego, CA, USA), and a different sample-specific multiplex adapter. We next pooled the libraries together, loaded the pool onto a single MiSeq flow cell (Ilumina Inc.), and sequenced the libraries using a MiSeq sequencer (Illumina Inc.) operated by the Genomic Diversity Center at ETH Zürich (Zürich, Switzerland.) We performed paired-end 150-cycle sequencing with the MiSeq Reagent Kit (version 2) (Illumina Inc.). All of the sequence reads are publically available in the NCBI Sequence Read Archive (http://www.ncbi.nlm.nih.gov/sra) under Bioproject ID number SUB2512304.

We analyzed the resulting sequence reads by first binning the raw sequence reads into libraries using the automated run protocol on the MiSeq sequencer (Illumina Inc.). We then performed quality control with FastQC version 0.10.1 and quality filtering using PrinSeq Lite version 0.20.4 software [38]. The parameters used for quality filtering are as follows: out_format, 3; min_qual_mean, 28; min_len, 50; range_gc 15–85; ns_max_n, 1; derep, 14; derep_min, 2; trim_ns_left, 1; trim_ns_right, 1; trim_qual_left, 28; trim_qual_right, 28; trim_left, 1. In summary, we trimmed or discarded all sequence reads with mean quality scores below 28 or had ambiguous nucleotides. We further discarded all sequence reads that were shorter than 50 bases. We finally applied the breseq pipeline (version 0.24rc1) and the utility program gdtools [39, 40] to predict genetic changes between each evolved mutualistic partner and its corresponding ancestral mutualistic partner. This included nucleotide polymorphisms, deletions, insertions, and multiplications. We used the genome sequence of *E. coli* K-12 MG1655 [41] as a reference for the mapping and assembly of the sequence reads. The parameters used for calling genetic changes are as follows: reference, NC_000913; base-quality-cutoff, 15; require-match-length, 30.

### Crossing experiment

We randomly selected the B2 mutualistic co-culture to test whether one or more of the evolved mutualistic partners were responsible for the evolution of cell aggregation. Based on colony morphology, fluorescent protein production, and genome analyses, we identified one evolved mutualistic partner of strain BW25113 (∆*proC*) and two different evolved mutualistic partners of strain BW25113 (∆*trpC*) within the B2 mutualistic co-culture immediately before the fourteenth transfer of experimental evolution (see Additional file 2). We first streaked each of the evolved mutualistic partners and their corresponding ancestral mutualistic partners from the archived isolate samples onto different LB agar plates that were supplemented with 10 μg ml^−1^ gentamycin and 1 mM IPTG. We then picked one colony from each LB agar plate, inoculated each colony into a different test tube containing 2.7 ml of MM supplemented with 300 μl of liquid LB medium, and incubated the test tubes for 24 h at 37 °C with continuous shaking (220 r. p. m.). After reaching stationary phase, we washed and suspended the cells in water containing 9 g L^−1^ NaCl as described above for the evolution experiment. We next prepared different binary (50:50 ratio based on OD_600_ measurements) or ternary (33:33:33 ratio based on OD_600_ measurements) mixes of different evolved and ancestral mutualistic partners as described in the results section. We finally inoculated 300 μl of each mix into replicated test tubes containing 2.7 ml of MM that was supplemented with 1 mM IPTG but not with amino acids. The initial OD_600_ of each mutualistic co- or tri-culture was approximately 0.12. After incubating the mutualistic co- or tri-cultures for 7 days at 37 °C with continuous shaking (200 r. p. m.), we measured the OD_600_ of each co-or tri-culture and quantified the magnitude of cell aggregation as described above.

## Results and discussion

### Experimental creation of a passive mutualistic interaction

We created a passive and obligate mutualistic interaction by inoculating pairs of the BW25113 (∆*proC*) and BW25113 (∆*trpC*) mutualistic partners (Table 1) together into MM that was not supplemented with amino acids. We found that the mutualistic partners could grow when they were inoculated together but could not grow when they were inoculated in isolation, thus demonstrating that we could indeed create the expected passive mutualistic interaction. We use the term ‘passive’ because the interaction emerges spontaneously when inoculating the two auxotrophic strains together that were neither engineered (e.g., as in [42]) nor evolved to actively excrete the amino acid that the other strain requires. We further tested whether access to these two amino acids limits the growth of the mutualistic co-cultures. We found that the co-cultures reached stationary phase approximately four-times more rapidly when we provided exogenous supplements of the required amino acids (50 mg L^−1^ L-proline and 20 mg L^−1^ L-tryptophan) (< 18 h) than when we did not (> 3.5 days), thus verifying that the supply of the required amino acids did indeed limit the growth of the mutualistic co-cultures.

### Evolutionary dynamics

We next investigated the evolutionary dynamics of the mutualistic co-cultures. We performed an evolution experiment by serially transferring the 16 replicated mutualistic co-cultures every 3.5 days into fresh MM that was not supplemented with amino acids, corresponding to 8 weeks of experimental evolution. Mutualistic co-cultures A1-A8 consisted of the *egfp*-expressing BW25113 (∆*proC*) and *echerry*-expressing BW25113 (∆*trpC*) mutualistic partners while mutualistic co-cultures B1 to B8 consisted of the *echerry*-expressing BW25113 (∆*proC*) and *egfp*-expressing BW25113 (∆*trpC*) mutualistic partners. We used two different combinations of fluorescent protein-encoding genes for two reasons. First, it allowed us to assess whether differences in the fluorescent protein-encoding genes themselves affect the outcome of the evolution experiment. Second, it allowed us to monitor for cross-contamination among the mutualistic co-cultures during the evolution experiment, which we never detected. We measured the OD_600_ of each mutualistic co-culture immediately before each subsequent transfer as a proxy of total cell numbers.

**Fig. 2:**
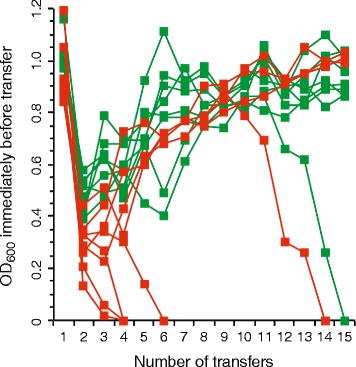
Experimental evolution of the mutualistic consortia. Sixteen replicate mutualistic consortia were assembled from the *egfp*-expressing BW25113 (∆*proC*) and *echerry*-expressing BW25113 (∆*trpC*) mutualistic partners (red symbols) or from the echerry-expressing BW25113 (∆*proC*) and *egfp*-expressing BW25113 (∆*trpC*) mutualistic partners (green symbols). Total cell numbers were then measured as OD_600_ and tracked over time. After the mutualistic interaction was imposed, ten consortia improved in growth performance over evolutionary time while six consortia went extinct, suggesting that different consortia undergo different evolutionary trajectories

We observed three qualitatively different evolutionary dynamics. For all 16 mutualistic co-cultures, the OD_600_ decreased by about 2.3-fold between the first and second transfers (Mann-Whitney U test, two-sided *P* = 1.5 × 10^−6^), indicating a substantial reduction in total cell numbers. Thereafter, however, the mutualistic co-cultures exhibited different dynamics. For three of the mutualistic co-cultures (co-cultures B4, B5, and B6), the OD_600_ continued to decrease and fell below detection levels at the fourth transfer (Fig. 2), indicating a persistent reduction in total cell numbers and eventual extinction. For another three of the mutualistic co-cultures (co-cultures A5, B3, and B8), the OD_600_ increased after the second transfer but then decreased again and fell below detection levels at the sixth, fourteenth, or fifteenth transfer (Fig. 2), indicating an initial increase in total cell numbers followed by a rapid shift in growth dynamics and eventual extinction. Finally, for the remaining ten mutualistic co-cultures, the OD_600_ increased nearly continuously and significantly by about 2.2-fold between the second and final transfers (Mann-Whitney U test, two-sided *P* = 1.8 × 10^−4^), demonstrating a progressive and substantial increase in total cell numbers and avoidance of extinction. These results are qualitatively consistent with a previous evolution experiment that imposed a mutualistic interaction between a methanogenic and a sulfate-reducing microorganism [43]. In that experiment, the authors similarly found that some mutualistic co-cultures went extinct while others progressively increased in total cell numbers and avoided extinction.

One limitation of our analysis above is our use of OD_600_ measures as proxy measures of total cell numbers, as cell aggregation can prevent a linear correspondence between OD_600_ and total cell numbers. In general, however, cell aggregation tends to decrease OD_600_. Thus, the fact that we observed a general increase in OD_600_ over evolutionary time as cell aggregation emerged suggests that total cell numbers also increased over the same evolutionary time. The increase in total cell numbers, however, should be interpreted as a qualitative observation rather than a quantitative measure.

### Evolution of cell aggregation

**Fig. 3:**
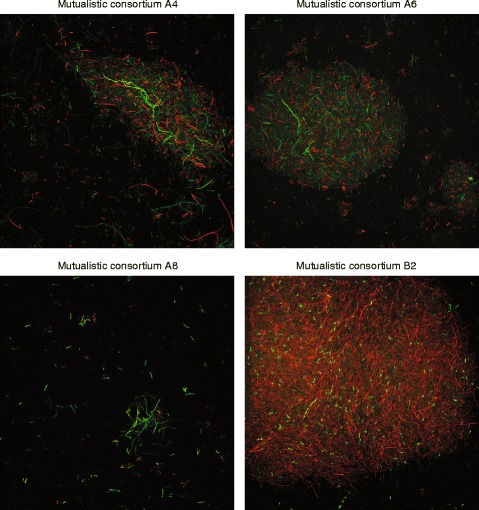
Representative images of cell aggregation within the mutualistic consortia. Images were obtained immediately before the fourteenth transfer of experimental evolution. For the A4, A6, and A8 consortia, green cells are the *egfp*-expressing BW25113 (∆*proC*) mutualistic partner and red cells are the *echerry*-expressing BW25113 (∆*trpC*) mutualistic partner. For the B2 consortium, red cells are the *echerry*-expressing BW25113 (∆*proC*) mutualistic partner and green cells are the *egfp*-expressing BW25113 (∆*trpC*) mutualistic partner. Note that all cell aggregates contained both mutualistic partners, but the qualitative structure and organization of the cell aggregates varied across the different consortia

**Fig. 4:**
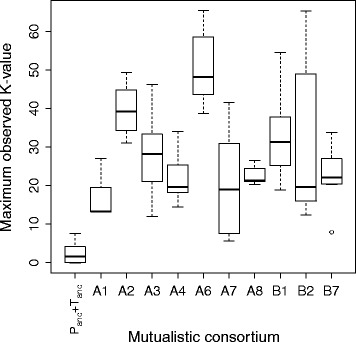
Magnitude of cell aggregation within the ancestral and evolved mutualistic consortia. Cell aggregation was measured as the maximum observed K-value. Definitions: P_anc_, ancestral strain of BW25113 (∆*proC*); T_anc_, ancestral strain of BW25113 (∆*trpC*). The data are presented as Tukey box plots. All the mutualistic consortia had larger K-values than the ancestral consortia, indicating an increase in cell aggregation over the course of the evolution experiment

We next examined whether the created passive mutualistic interaction promotes the subsequent evolution of cell aggregation. To accomplish this, we used a confocal microscope to analyze the spatial structure of the mutualistic co-cultures that avoided extinction immediately before the fourteenth transfer. We found that the cells within all of these mutualistic co-cultures were significantly more aggregated together than were the cells within their corresponding ancestral mutualistic co-culture (Fig. 3). All observed aggregates contained mixtures of both mutualistic partners. All of the evolved mutualistic co-cultures had significantly larger maximum observed K-values (which we used as a measure of cell aggregation as described in the Materials and Methods) than did their corresponding ancestral mutualistic co-cultures (Fig. 4 and Table 2; Mann-Whitney U test, two-sided *P* ≤ 0.0021). This indicates that cells within the evolved mutualistic co-cultures were more heterogeneously distributed in space via cell aggregation than were the cells within their corresponding ancestral mutualistic co-culture. In general, aggregation was qualitatively similar within lineages but varied across lineages (Fig. 3). We further found that cell aggregation only evolved in co-cultures consisting of strains coupled by the passive mutualistic interaction. Spatial structure never evolved when we prevented the mutualistic interaction from establishing by growing the ancestral mutualistic partners together in LB liquid medium or in MM supplemented with the required amino acids (note that the MM contained gentamycin and IPTG and the strains contained their respective plasmids in these controls). Finally, there was no statistically detectable effect of the combination of fluorescent proteins used to mark each strain on the maximum observed K-values reported in Fig. 4 (i.e. there was no difference whether the ∆*trpC* or ∆*proC* mutant expressed *egfp* or *echerry*) (Mann-Whitney U-test, two-sided *P* > 0.05). Taken together, our results demonstrate that the creation of the passive mutualistic interaction was necessary for and promoted the subsequent evolution of cell aggregation.

**Table 2:** Comparison of the K-values for the test and ancestral mutualistic consortia

Experiment	^a^Mutualistic consortium	^b^ *P*
Evolution experiment	A1	**0.0013**
	A2	**0.0013**
	A3	**0.0013**
	A4	**0.0013**
	A6	**0.0013**
	A7	**0.0021**
	A8	**0.0013**
	B1	**0.0003**
	B2	**0.0003**
	B7	**0.0004**
^c,d^Crossing experiment	P_anc_ + T_evol,a_	0.015
(mutualistic consortium B2)	P_anc_ + T_evol,b_	0.028
	P_evol_ + T_anc_	0.17
	P_evol_ + T_evol,a_	**0.0002**
	P_evol_ + T_evol,b_	**0.0009**
	P_evol_ + T_evol,a_ + T_evol,b_	**0.0002**

^a^Only those consortia that avoided extinction were analyzed. ^b^Bold font: statistically significant differences between the test and ancestral mutualistic consortia (*P* < 0.01). ^c^Definitions: P_anc_, ancestral strain of BW25113 (∆*proC*); T_anc_, ancestral strain of BW25113 (∆*trpC*); P_evol_, evolved strain of BW25113 (∆*proC*); T_evol,a_, evolved strain of BW25113 (∆*trpC*); T_evol,b_, evolved strain of BW25113 (∆*trpC*). ^d^T_evol,a_ and T_evol,b_ are two genetically different strains of BW25113 (∆*trpC*) that emerged within the B2 mutualistic consortium

One alternative explanation is that the cell aggregates consisted of dead cells rather than viable cells. To test this, we routinely plated the cultures onto agar plates. After incubation, we always observed individual colonies that expressed both fluorescent proteins (i.e., they contained mixtures of cells that express *gfp* or *echerry*). This indicates that the mutualistic partners were indeed physically attached to each other and that cells within the aggregates were viable. The cell aggregation phenotype therefore clearly contributed towards population-level phenotype. However, as shown in Fig. 3, individual planktonic cells remained. Thus, while aggregation significantly contributed to population-level phenotype, it remained incomplete.

While we observed the emergence of cell aggregation, we note that our results do not suggest that the emergence of cell aggregation was directly caused by the mutualistic interaction itself. This could occur via mechanisms such as specific partner recognition, where each mutualistic type aggregates with its partner. However, such adaptations would likely require long evolutionary times. Instead, the emergence of cell aggregation in our study is likely an indirect consequence of the small amounts of amino acids that leak or are released from the mutualistic partners. In other words, the mutualistic interaction creates an environment with low concentrations of amino acids, which then promotes the evolution of non-specific cell aggregation. Regardless, non-specific cell aggregation might then set the stage for further evolutionary changes, such as the emergence of partner recognition and specific attachment between the different mutualistic partners.

### Genetic changes during emergence of cell aggregation

We investigated the genetic changes that occurred during the evolution experiment. The goal here was not to identify the specific genetic changes that cause cell aggregation, but rather to investigate whether each lineage accumulated similar or different genetic changes during the acquisition of the cell aggregation phenotype. This then allows us to hypothesize whether there is a single or multiple evolutionary pathways to cell aggregation. To accomplish this, we reconstructed the ancestral and evolved mutualistic co-cultures from isolates. We first grew each ancestral or evolved mutualistic partner on amino acid-rich LB agar plates, assembled the mutualistic partners together into mutualistic co-cultures, and inoculated the co-cultures into MM that was not supplemented with amino acids. We found that the reconstructed evolved mutualistic co-cultures formed cell aggregates after 7 days of incubation while the ancestral mutualistic co-cultures did not (i.e., the ancestral cells were completely planktonic in microscopy images). Thus, cell aggregation was heritable and therefore likely had a genetic basis.

We next sequenced the genomes of the ancestral and evolved isolates and identified genetic changes in the evolved mutualistic partners that might have caused the observed emergence of cell aggregation. We provide a complete list of all the observed genetic changes in Additional file 2. We excluded mutualistic co-culture A2 from the analysis because we could not separate the mutualistic partners into individual cells (i.e., they continued to form mixed-strain colonies after repeated streaking on LB agar plates, indicating very strong aggregation). Each evolved mutualistic co-culture contained a mutualistic partner that fixed at least one genetic change located within or immediately upstream of a gene known to be involved with biofilm formation (Table 3). However, as opposed to comparable experimental evolution studies and analytical methods [40, 44–47], we observed relatively limited evolutionary parallelism of the genetic changes. More than 75% (13/17) of the genes or upstream regions that contained genetic changes were changed in only one co-culture (Table 3). Thus, there appears to be a large number of possible genetic targets that could result in the evolution of cell aggregation. This is not unexpected given the large number of gene products required for the regulation, initiation, development and maturation of *E. coli* biofilms [48–50].

**Table 3:** Genetic changes within or upstream of genes that have experimentally verified roles in *E. coli* biofilm formation in other studies

^a^Mutualistic consortium	^b^Evolved mutualistic partner	Mutation type	Gene(s) or intergenic region	Role of gene(s) in *E. coli* biofilm formation	Reference
A1	BW25113 (∆*proC*)	non-synonymous point mutation, A- > G	*spoT* ^c^	c-di-GMP regulation	[51, 52]
A3	BW25113 (∆*proC*)	Δ16 bp, intergenic region	upstream of *flhD*	motility regulation	[57, 58]
	BW25113 (∆*trpC*)_a_	Δ1 bp, coding region	*hdfR*	motility regulation	[58, 59]
	BW25113 (∆*trpC*)_b_	Δ1 bp, coding region	*hdfR*	motility regulation	[58, 59]
A4	BW25113 (∆*proC*)	non-synonymous point mutation, G- > A	*flhC*	motility regulation	[60]
	BW25113 (∆*trpC*)_a_	Δ627 bp, coding and intergenic regions	*spoT* ^c^-*trmH*	c-di-GMP regulation	[51, 52]
	BW25113 (∆*trpC*)_b_	Δ627 bp, coding and intergenic regions	*spoT* ^c^-*trmH*	c-di-GMP regulation	[51, 52]
A6	BW25113 (∆*trpC*)	non-synonymous point mutation, C- > T	*glmU* ^c^	extracellular matrix	[53, 54]
	BW25113 (∆*trpC*)	point mutation, intergenic region	upstream of *yqcC*	biofilm maturation	[61]
A7	BW25113 (∆*trpC*)_a_	Δ15 bp, coding region	*spoT* ^c^	c-di-GMP regulation	[51, 52]
	BW25113 (∆*trpC*)_b_	Δ15 bp, coding region	*spoT* ^c^	c-di-GMP regulation	[51, 52]
	BW25113 (∆*trpC*)_c_	Δ15 bp, coding region	*spoT* ^c^	c-di-GMP regulation	[51, 52]
A8	BW25113 (∆*proC*)	Δ3 bp, coding region	*glmU* ^c^	extracellular matrix	[53, 54]
	BW25113 (∆*proC*)	Δ3 bp, coding region	*gspA*	biofilm maturation	[62]
	BW25113 (∆*proC*)	Δ3 bp, coding region	*rcsF*	EPS regulation	[63, 64]
	BW25113 (∆*trpC*)_c_	Δ6 bp, coding region	*bamA*	extracellular matrix	[65, 66]
B1	BW25113 (∆*trpC*)	Δ11 bp, coding region	*nlpI*	extracellular matrix	[67]
B2	BW25113 (∆*proC*)	Δ10 bp, intergenic region	upstream of *bluF* and *ycg*	EPS regulation	[61, 62]
	BW25113 (∆*proC*)	Δ5 bp, coding region	*dksA*	c-di-GMP regulation	[61, 63, 68]
	BW25113 (∆*proC*)	Δ3 bp, intergenic region	upstream of *yliE*	c-di-GMP regulation	[52]
	BW25113 (∆*proC*)	113 bp duplication of coding region	*yeaP*	fimbriae regulation	[69]
	BW25113 (∆*trpC*)_a_	non-synonymous point mutation, G- > T	*gpp*	c-di-GMP regulation	[52, 70]
B7	BW25113 (∆*proC*)	Δ1 bp, coding region	*bluR*	EPS regulation	[56]

^a^Only those consortia that avoided extinction were analyzed. ^b^Subscripts indicate that more than one clone of that mutualistic partner was sequenced from the corresponding mutualistic consortium. ^c^Genetic changes in *spoT* and *glmU* have been observed in other studies where cell aggregation did not emerge

While we observed limited evolutionary parallelism in the genetic changes, a few genes or upstream regions were changed in mutualistic partners from more than one co-culture. Genetic changes in *spoT* or its upstream region occurred in mutualistic partners from three co-cultures (Table 3). *spoT* affects biofilm formation by modifying levels of (p) ppGpp [51, 52] and, under certain conditions, the inactivation of *spoT* can enhance biofilm formation [51]. Genetic changes in *glmU* occurred in mutualistic partners from two co-cultures (Table 3). *glmU* affects biofilm formation by controlling the biosynthesis of surface adhesion molecules [53, 54]. We note here, however, that genetic changes in *spoT* and *glmU* have been reported in other evolution experiments where cell aggregation did not emerge [40], and that further molecular work would therefore be required to test their role here. Genetic changes in three flagellar genes (*flhC*, *flhD*, and *hdfR*) occurred in mutualistic partners from two co-cultures (Table 3). These genes affect biofilm formation and surface attachment by regulating the biosynthesis of flagella [55]. Finally, genetic changes in three other genes or upstream regions (*bluR*, *bluF, ycgG)* occurred in mutualistic partners from two co-cultures (Table 3). These genes affect biofilm formation by activating the Rcs system, which regulates the biosynthesis of surface adhesion molecules and curli fimbre [56]. We did observe some mutations in *lacI,* which is involved with the transcriptional regulation of the fluorescent proteins. These mutations were not unique to one mutualistic partner or another, and they are therefore unlikely to have created confounding factors that compromise our main conclusions.

### Both mutualistic partners are required for cell aggregation

**Fig. 5:**
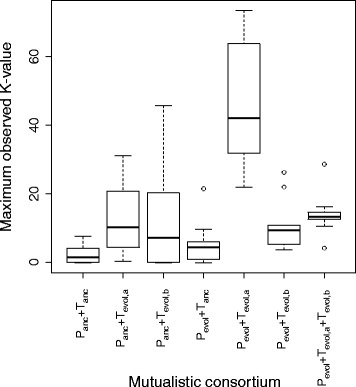
Magnitude of cell aggregation for different combinations of ancestral and evolved mutualistic partners from the B2 mutualistic consortium. Cell aggregation was measured as the maximum observed K-value. Definitions: P_anc_, ancestral strain of BW25113 (∆*proC*); T_anc_, ancestral strain of BW25113 (∆*trpC*); P_evol_, evolved strain of BW25113 (∆*proC*); T_evol,a_, evolved strain of BW25113 (∆*trpC*); T_evol,b_, evolved strain of BW25113 (∆*trpC*). T_evol,a_ and T_evol,b_ are two genetically different strains of BW25113 (∆*trpC*) that emerged within the B2 mutualistic consortium. The data are presented as Tukey box plots. Note that the K-values (and thus cell aggregation) were the largest for pairs of evolved mutualistic partners, suggesting that genetic changes in both partners contributed to the observed cell aggregation

The genome sequences demonstrate that both mutualistic partners could acquire mutations in biofilm-related genes or in regions immediately upstream of those genes. This observation then leads to the following hypothesis: both mutualistic partners could contribute towards the observed evolution of cell aggregation. To test this hypothesis, we performed a crossing experiment with the B2 mutualistic co-culture. Based on colony morphology and genome sequences, we determined that this mutualistic co-culture had one evolved mutualistic partner of BW25113 (∆*proC*) (designated as P_evol_) and two different evolved mutualistic partners of BW25113 (∆*trpC*) (designated as T_evol,a_ and T_evol,b_) (see Additional file 2). T_evol,a_ contains one genetic change not present in T_evol,b_ while T_evol,b_ contains seven genetic changes not present in T_evol,a_ (see Additional file 2). We first grew each mutualistic partner in isolation and then mixed the evolved mutualistic partners together or mixed each evolved mutualistic partner with its corresponding ancestral mutualistic partner of BW25113 (∆*proC*) (designated as P_anc_) or BW25113 (∆*trpC*) (designated as T_anc_). We finally quantified cell aggregation of the resulting mutualistic consortia using a confocal microscope (Fig. 5). We acknowledge here that we only performed these experiments for one lineage; the results may therefore be lineage-specific and we cannot make general statements. Instead, our objective here was to investigate a single lineage in order to provide initial insight into the observed phenomena and set the stage for future investigations (e.g., to investigate evolutionary parallelism, etc.).

We found that heritable changes in both evolved mutualistic partners are required to maximize cell aggregation. Mutualistic consortia of P_anc_ and T_evol,a_ or T_evol,b_ produced significantly more cell aggregation than mutualistic consortia of P_anc_ and T_anc_ (Fig. 5 and Table 2; Mann-Whitney U test, two-sided *P* < 0.05). In contrast, mutualistic consortia of P_evol_ and T_anc_ did not form significantly more cell aggregation than mutualistic consortia of P_anc_ and T_anc_ (Fig. 5 and Table 2; Mann-Whitney U test, two-sided *P* > 0.05). However, mutualistic consortia of P_evol_ and T_evol,a_ or T_evol,b_ produced the most significant increase in cell aggregation (Fig. 5 and Table 2; Mann-Whitney U test, two-sided *P* < 0.05). Thus, within the B2 mutualistic consortium, the most substantial increase in cell aggregation occurred when mutations in both mutualistic partners were present together within the consortium.

### Only one mutualistic partner contributes towards increased cell numbers

**Fig. 6:**
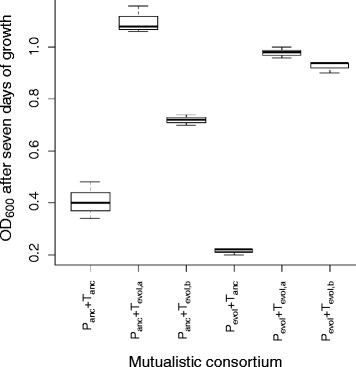
Total cell numbers for different combinations of ancestral and evolved mutualistic partners from the B2 mutualistic consortium. Total cell numbers were measured as the OD_600_. Definitions: P_anc_, ancestral strain of BW25113 (∆*proC*); T_anc_, ancestral strain of BW25113 (∆*trpC*); P_evol_, evolved strain of BW25113 (∆*proC*); T_evol,a_, evolved strain of BW25113 (∆*trpC*); T_evol,b_, evolved strain of BW25113 (∆*trpC*). T_evol,a_ and T_evol,b_ are two genetically different strains of BW25113 (∆*trpC*) that emerged within the B2 mutualistic consortium. The data are presented as Tukey box plots. Note that the OD_600_ measurements (and thus cell density) were the largest for consortia containing the evolved ∆*trpC* partner, regardless of the evolutionary history of the ∆*proC* partner. Thus, mutations in only the ∆*trpC* partner are sufficient to explain the increase in OD_600_ over the course of the evolution experiment

We next asked whether both evolved mutualistic partners contribute towards increased cell numbers of the mutualistic co-cultures (measured as the OD_600_ after 7 days). To test this, we performed the same crossing experiment with the B2 mutualistic consortium as described above. We found that mutations in only one evolved mutualistic partner contributed towards increased cell numbers. Mutualistic co-cultures of P_anc_ and T_evol,a_ or T_evol,b_ had significantly higher OD_600_ than mutualistic co-cultures of P_anc_ and T_anc_ and could account for all or a substantial portion of the observed increase in growth of the evolved mutualistic co-culture (Fig. 6; Welch test, two-sided *P* < 0.02). In contrast, mutualistic co-cultures of P_evol_ and T_anc_ had significantly lower OD_600_ than mutualistic co-cultures of P_anc_ and T_anc_ (Fig. 6; Welch test, two-sided *P* = 0.0382). Thus, genetic changes in the P_evol_ mutualistic partner resulted in a growth defect when combined with its ancestral mutualistic partner. This suggests that the evolutionary changes in P_evol_ were acquired in direct response to evolutionary changes in T_evol,a_ or T_evol,b,_ and are therefore a result of co-evolutionary dynamics between the mutualistic partners. Thus, within the B2 mutualistic co-culture, mutations in only one mutualistic partner was sufficient to explain the observed increase in cell numbers – but not necessarily the growth rate – of the B2 mutualistic co-culture over evolutionary time. After these initial mutants emerge, this may then set the stage for future mutants to emerge that reinforce the mutualistic interaction. We believe these dynamics are generalizable, in that the origin of any mutualistic interaction must necessarily emerge via single genetic changes. The dynamics with respect to the further strengthening of the mutualistic interaction, where mutants of both cell-types increase in frequency, would require longer evolutionary times than investigated in this study. We acknowledge here that we are not investigating the effects of individual genetic changes, which would require introducing those genetic changes into the ancestral background. It is possible that the presence of multiple genetic changes may be critically important for our observations, such as reciprocal adaptations between the mutualistic partners.

## Conclusions

We propose that the scenario we investigated here might be applicable to different types of organisms and interactions: for many organisms, proximity to members of the same or another genotype might be advantageous, for example because these other individuals provide resources or protection. This is expected to impose selection for biological traits that ensure proximity, through physical attachment, behavior, or by other means. This would lead to a situation that is equivalent to spatial structure; that is, a situation where individuals within or between genotypes are associated with each other for extended periods of time, as assumed in the “partner fidelity” model [24]. According to this scenario, mutually beneficial interactions within and between organisms are expected to evolve more readily than often assumed, because the organisms themselves can readily generate the spatial structure that is necessary for these beneficial interactions to emerge and be stable. While this scenario is consistent with our results, a conclusive test would require tracking the spatial positioning of individual cells over evolutionary time, which is not possible with our current data set. However, recent developments in analytical microbiology should now allow for such tracking and for explicit tests of this hypothesis.

## Additional files

Additional file 1: Table of oligonucelotide PCR primers used in this study. (DOCX 52 kb)

Additional file 2: Genetic changes observed after experimental evolution in this study. (XLSX 62 kb)
