# Supplementary material for: A passive mutualistic interaction promotes the evolution of spatial structure within microbial populations
Source: BMC Evol Biol. 2017 Apr 24;17:106. doi: 10.1186/s12862-017-0950-y (PMC5402672; doi:10.1186/s12862-017-0950-y)
Supplement: Supplementary file 1 — Table of oligonucelotide PCR primers used in this study. (DOCX 52 kb) [file 12862_2017_950_MOESM1_ESM.docx]

**Additional file 1.** Oligonucelotide PCR primers used for amplifying and cloning the *egfp* or *echerry* gene into the pUC18T-mini-Tn7T-LAC-Gm plasmid.

| **Gene** | **Direction** | **^a^Primer sequence (5’-3’)** | **Restriction site** |
| --- | --- | --- | --- |
| *egfp* | forward | CGCGGATCCTGATTAACTTTATAAGGAGGAAAAACATATGAGTAAAGGAGAAGAACTTTTCACT | *Bam*HI |
|  | reverse | CGGGGTACCTTTGTATAGTTCATCCATGCCATG | *Kpn*I |
| *echerry* | forward | CGCGGATCCTGATTAACTTTATAAGGAGGAAAAACATATGGTTTCCAAGGGCGAG | *Bam*HI |
|  | reverse | CGGGGTACCTTATTTGTACAGCTCATCCATGC | *Kpn*I |

^a^Red: leader sequence. Blue: restriction sequence. Green: ribosomal binding sequence. Black: target-specific sequence.
